# Results and summary of voting among the audience during presentation and discussion of Medullary Thyroid Carcinoma clinical guidelines prepared by American Thyroid Association

**DOI:** 10.1186/1756-6614-6-S1-S12

**Published:** 2013-03-14

**Authors:** Ulla Feldt-Rasmussen, Folke Soderstrom

**Affiliations:** 1 Department of Medical Endocrinology, National University Hospital, Copenhagen University, Blegdamsvej 9, DK-2100 Copenhagen, Denmark; 2 Hovtek AB, Tellusvagen 9 , 191 47 Sollentuna, Denmark

## Abstract

The one-day ETA-CRN meeting, preceding the ETA meeting in Lisbon, was planned in advance to provide a thorough assessment of the European response to the published American Thyroid Association MTC guidelines. During the meeting, following each of the European speakers, a series of questions, related to the specific aspect, were presented to the audience. The responses from the audience were collected by an AudioResponseSystem (ARS voting system). The results of the voting showed in summary that European expert opinion leaders and an audience of specialists in treatment of Medullary Carcinoma welcomes the American Guidelines on the management of MTC, but simultaneously only partially agrees with some of the statements in the guidelines.

## Introduction

The one-day ETA-CRN meeting, preceding the ETA meeting in Lisbon, was planned in advance to provide a thorough assessment of the European response to the published American Thyroid Association MTC guidelines [1]. This was done by different approaches:

1. A scientific programme was organised starting with a presentation of the guidelines by Richard Kloos, followed by European speakers, presenting various aspects of the guidelines.

2. Before the meeting, following an extensive on-line discussion among European experts, a series of questions in relation to the American guidelines were sent to all presenters, asking for his/her opinion prior to the meeting and the result were presented in Jarzab B et al. Presentation of points of general discussion and voting among the speakers [2].

3. During the meeting, following each of the European speakers, a series of questions, related to the specific aspect, were presented to the audience. The responses from the audience were collected by an AudioResponseSystem (ARS voting system). The results of this voting are summarised below and the full details presented in questions and answers by the Participants (Additional file 1).

## Summary of voting results

The initial questions were asked to identify the individual members of the audience, and demonstrated that the audience was 81% Europeans and 83% doctors in clinical disciplines. The majority of the participants were specialists in medical endocrinology (62%), oncology (11%) or endocrine/oncological surgery (13%), and most worked in an academic university hospital (72%).

Only 18% were either not a clinical doctor or a doctor not treating patients with MTC. The remaining 82% were equally distributed among those treating >50, between 10 and 50 and below 10 patients, respectively.

Above basic questions were asked after each break in order to correctly identify the individual person’s voting results with that person’s background. The main reason for that was to try to correlate responses to the background e.g. differences in response between Europeans and others, between clinicians and basic scientists, between clinicians in medical endocrinology vs oncology or surgery, between clinicians in university hospitals vs local hospitals or private praxis etc. This subgrouping was eventually not performed due to the small numbers in some of the groups and thus a dominance of others. The results shown in Additional file 1 only display the first background voting.

The summary of results is thus based on the audience as a whole without any subgroups, with some differences in numbers for each question and at different parts of the meeting.

The number of actively participating voters is provided for each question. There were 138 participants. A total of 39 questions were asked with between 73 and 127 responders.

The present summary of results will mainly highlight the answers, that demonstrated a discrepancy i.e. when the European opinion diverged from the American guidelines.

Sixty% of the audience was in favour of measuring plasma calcitonin in all patients referred for nodular thyroid disease, while 19% would like to see a cost-effectiveness study, and 21% agreed with the American guidelines (Q1). Less than half agreed with the ATA guidelines on a fixed cut-off for serum calcitonin, while the majority would require local laboratory references (Q2). Sixty-six% were in favour of performing a pentagastrin stimulation test in all patients whose serum calcitonin was either low or in the grey zone. Few agreed with the ATA guidelines (27%)(Q3).

The majority (45%) found that completion thyroidectomy is always indicated after an unexpected diagnosis of MTC, and should be completed by at least central LND, even if postoperative calcitonin was normal, while 29% voted for size of the process to decide the procedure, in disagreement with the ATA recommendations (Q5).

Question Q7 considered the indications for lateral lymph node surgery and 79% of responders disagreed with the ATA guidelines, which recommended against prophylactic lateral node dissection irrespective of the central neck lymph node status. Among them, 46% agreed with a minority opinion of the American Task Force favouring prophylactic (elective) lateral neck dissection when lymph node metastases were present in the adjacent paratracheal central compartment. However, this question was quite difficult to be answered and, after questions from the audience, it was decided by the audience to repeat the voting with an additional alternative response: (Lateral) lymph node dissection should be performed if increased basal or stimulated CT was found in a patient with (after) central LND. In the repeated voting 32% voted for the added alternative and in total, 86% of the answers disagreed with the ATA guidelines. In Q8 there was also a disagreement, since approx. half of responders found a stimulation test to be more sensitive than just a basal serum calcitonin measurement without pentagastrin stimulation. For a patient with measurable Ct postoperatively, a significant part of responders (38%) supported very distinctly the central CND, if previously not done. Other 54% agreed with the ATA guideline, also supporting this intervention but with more caution.

Questions Q9 and Q11-Q18 were related to the staging and treatment of advanced MTC, generally with significant agreement with the ATA Guidelines among responders, ranging between 68% and 84%.

In the third part of the meeting, the guidelines related to DNA diagnosis and hereditary MTC were discussed (Q19-Q39). Q 21 illustrates some of the levels of disagreement.

Only one third of participants agreed with the guidelines on the desirable extent of RET diagnostics.

This was also the case for 55% of responders, who did not agree on the narrowing of indications for early testing for MEN 2B, being such a debilitating disease. According to the European opinion, a RET mutation analysis should be offered early (Q26). Thirty-seven % of the European responders were furthermore in favour of using stimulated serum calcitonin levels to decide on the time of prophylactic surgery (Q29).

Overall a median of 53% of the audience (Range 14 – 84%) agreed with the American Guidelines, and in general between 3 and 9% had no opinion on the specific question asked. This left roughly 40% in disagreement with the guidelines.

Among the experts asked ahead of the meeting (data not shown), the disagreement was even stronger than that of the audience during the meeting, in particular if leaving out one expert, who participated in the Task Force. These results were left out, since they were considered biased due to the selection of the experts.

## Conclusions

European expert opinion leaders and an audience of specialists in treatment of Medullary Carcinoma welcomes the American Guidelines on the management of MTC, but simultaneously only partially agrees with some of the expressed statements. The results of the survey prior to the meeting were biased in that the presenters were selected for presenting the results, but the audience was present upon open invitation through scientific channels. Notwithstanding the evidence based guidelines, their final acceptance requires unrestricted discussion and consideration of the differences in clinical practice and experience between countries.

These discussions and results subsequently formed the basis for establishing a task force within the ETA, and consequent publication of European guidelines mainly for the treatment aspects of metastatic MTC.

## Competing interests

No competing interests exist for me and my co-authors.

## Supplementary Material

Additional file 1 Annex 1: Questions and answers by the participants of the ETA-CRN Session on ATA MTC Guidelines, Lisbon. (Q indicates question; n indicates the number of voters for each question) Click here for file

## Declarations

This article has been published as part of *Thyroid Research* Volume 6 Supplement 1, 2013: European comments on Medullary Thyroid Cancer Management Guidelines of the American Thyroid Association. The full contents of the supplement are available online at http://www.thyroidresearchjournal.com/supplements/6/S1. Publication of this supplement has been supported by the European Thyroid Association-Cancer Research Network.

## References

[B1] American Thyroid Association Guidelines Task Force Kloos RT Eng C Evans DB Francis GL Gagel RF Gharib H Moley JF Pacini F Ringel MD Schlumberger M Wells SA Jr Medullary thyroid cancer: management guidelines of the American Thyroid Association Thyroid 2009 19 565 612 10.1089/thy.2008.0403 19469690

[B2] Jarzab B Krol A Hasse-Lazar K Jurecka-Lubieniecka B Presentation of points of general discussion and voting among the speakers Thyroid Research 2013 6 Suppl 1 S11 10.1186/1756-6614-6-S1-S11 PMC3599728 23514345

[B3] Schlumberger M Bastholt L Dralle H Jarzab B Pacini F Smit JWA 2012 European Thyroid Association Guidelines for Metastatic Medullary Thyroid Cancer Eur Thyroid J 2012 1 5 14 10.1159/000336977 PMC3821456 24782992

