# Supplementary material for: Results and summary of voting among the audience during presentation and discussion of Medullary Thyroid Carcinoma clinical guidelines prepared by American Thyroid Association
Source: Thyroid Res. 2013 Mar 14;6(Suppl 1):S12. doi: 10.1186/1756-6614-6-S1-S12 (PMC3599720; doi:10.1186/1756-6614-6-S1-S12)
Supplement: Additional file 1 — Annex 1: Questions and answers by the participants of the ETA-CRN Session on ATA MTC Guidelines, Lisbon. (Q indicates question; n indicates the number of voters for each question) [file 1756-6614-6-S1-S12-S1.doc]

**Additional file 1 – Annex 1: Questions and answers by the participants of the ETA-CRN Session on ATA MTC Guidelines, Lisbon. (Q indicates question; n indicates the number of voters for each question)**

Q A1 (n=113)

Is this your first time in Lisbon?

1. Yes 47%

2. No 53%

Q B1 (n=130)

Where do you come from?

1. Europe 81%

2. USA 5%

3. Other 14%

Q C1 (n=138)

What is your profession?

1. Medical Doctor 83%

2. Basic Scientist 8%

3. Student in clinical medicine 2%

4. Student in basic science 1%

5. Pharmaceutical Company 2%

6. Other 4%

Q D1(n=129)

If you are in clinical medicine, what is your Speciality?

1. Medical Endocrinology 62%

2. Oncology 11%

3. Endocrine/Oncological Surgery 13%

4. Paediatrics 0%

5. Nuclear Medicine 7%

6. Clinical Biochemistry 2%

7. Other 5%

Q E1 (n=134)

Where are you primarily working?

1. University Hospital 72%

2. Regional Hospital 13%

3. Smaller Hospital 2%

4. Private praxis 6%

5. Basic University 1%

6. Pharmaceutical Company 2%

7. Other 4%

Q 1 (n=120)

The ATA Guideline R52 defers the recommended approach to thyroid nodules, including fine needle aspiration biopsy and serum Ct testing, to the ATA Guideline that addresses thyroid nodules. The European consensus – endorsed by ETA - for management of patients with DTC

of the follicular epithelium recommends Ct testing in nodular disease.

What is your opinion?

1. I accept the ATA guideline in its full extent 21%

2. I agree with the European consensus on Ct estimation in all

patients with thyroid nodules, and perform it. 45%

3. I agree with the European consensus on Ct estimation in all patients with thyroid

nodules, but I am unable to perform it in all patients for financial reasons 15%

4. Obligatory Ct estimation needs better evaluation of its cost-effectiveness 19%

Q 2 (n=104)

The reference range of basal Ct has a very high interlaboratory variability and also a gender difference. Yet, the ATA guidelines refer to fixed cut-off levels.

Do you agree on this practice?

1. Yes. I prefer to have one reference range, with normal values ≤ 10ng/L 43%

2. No. Each laboratory should perform its own specific reference range and ROC

curves for cut-off limits 47%

3. I have no opinion on this matter 10%

Q 3 (n=114)

The ATA guideline R52 defines a basal or stimulated* serum Ct level > 100 ng/L,

as suspicious for MTC. What is you opinion on this?

1. I accept the ATA guideline in its full extent 27%

2. I agree that basal Ct >100 ng/L means a substantial risk of MCT. A pentagastrin

stimulated cut-off at 100ng/L should be recommended for the grey zone 10-100ng/L 49%

3. I agree with 2 but prefer to set the cut-off for stimulated Ct at 50 ng/L 13%

4. I agree with 2 but prefer to set the cut-off for stimulated Ct at 200 ng/L or even greater 4%

5. I have no opinion on this matter 7%

Q 4 (n=108)

Preoperative chest CT, neck CT, and 3 phase contrast enhanced multidetector liver CT

or contrast enhanced MRI is recommended for all patients with suspected MTC when

local lymph node metastases are detected (N1), or serum Ct is > 400 pg/ml.

What is you opinion on this?

1. I agree with the ATA guideline 62%

2. Above preoperative imaging is only indicated when serum Ct is larger than

1000-2000 ng/L. 14%

3. These examinations are obligatory in all cases except in case of prophylactic

thyroidectomy 13%

4. I have no opinion on this matter 11%

Q 5 (n=110)

In ATA R71-71, completion thyroidectomy may be postponed after hemithyroidectomy,

if unifocal intrathyroidal sporadic MTC, confined to the thyroid, no C-cell hyperplasia,

neg surgical margin, no suspicion of persistent disease on neck US and basal serum Ct

below upper reference limit > 2 months after surgery

1. I agree with the ATA guideline 29%

2. Completion thyroidectomy is always indicated after unexpected diagnosis of MTC,

and should be completed by at least central LND, even if postop Ct is normal 45%

3. The indication depends on the size of the primary tumour. The conditions listed in 2

is valid only if solitary infracentimetric MTC was found 20%

4. I have no opinion on this matter 5%

Q 6 (n=126)

The ATA R61 states “Patients with known or highly suspected MTC with no evidence of

advanced local invasion by the primary tumour, no evidence of cervical lymph node

metastases on physical examination and cervical US, and no evidence of distant

metastases should undergo total thyroidectomy and prophylactic central compartment

(level VI) neck dissection”. Do you agree on this matter?

1. Yes. Because the absence of any enlarged lymph node by ultrasound does not exclude

the presence of lymph node metastases in MTC. 80%

2. No. Prophylactic central lymph node dissection may not be necessary in very small

tumours detected by Ct screening 13%

3. I have no opinion on this matter 7%

Q 7 (n=120)

R62 states that if lymph node metastases are not detected by ultrasound, the elective lateral lymphadenectomy is not necessary. However:“A minority of the Task Force favoured

prophylactic lateral neck dissection when lymph node metastases were present in the

adjacent paratracheal central compartment”. What is your opinion on this statement?

1. If no enlarged lateral LN are detected, elective lateral lymph node dissection is not

obligatory in MTC, irrespective of the status of central neck lymph node 21%

2. With no enlarged lateral LN, elective lateral LND should be done only when lymph node metastases are present in the adjacent paratracheal central compartment 46%

3. Elective lateral lymph node dissection is always obligatory in MTC 29%

4. I have no opinion on this matter 4%

Q 7/2 (n=96)

R62 states that if lymph node metastases are not detected by ultrasound, the elective lateral lymphadenectomy is not necessary. However: “A minority of the Task Force favoured prophylactic lateral neck dissection when lymph node metastases were present in the

adjacent paratracheal central compartment”. What is your opinion on this statement?

1. If no enlarged lateral LN are detected, elective lateral lymph node dissection is not

obligatory in MTC, irrespective of the status of central neck lymph node 14%

2. With no enlarged lateral LN, elective lateral LND should be done only when lymph node metastases are present in the adjacent paratracheal central compartment 35%

3. Elective lateral lymph node dissection is always obligatory in MTC 16%

4. Lymphnode dissection should be performed if in patients with central LND increased

basal or stimulated CT is stated 32%

5. I have no opinion on this matter 3%

Q 8 (n=119)

The ATA guidelines R73-74 recommend postoperative follow-up based on Ct and CEA

estimation. Do you agree on this statement?

1. Yes 41%

2. No. Only basal Ct should be measured 11%

3. Stimulated Ct is more sensitive than basal Ct level and should be performed annually 14%

4. Pentagastrin test should be performed at first postoperative evaluation if basal Ct is low. 30%

5. I have no opinion on this matter 3%

Q 9 (n=120)

The ATA R75 guideline proposes a cut-off of <150 ng/L, below which postoperative

imaging may be limited to US only. R76 recommends that post-operative MTC patients with detectable serum Ct levels <150 ng/L may be considered for additional imaging (CT/MRI)

to serve as baseline examinations for future comparison even though these studies are

usually negative. Do you agree?

1. I agree with R75. Additional imaging can subsequently be implemented should the

serum Ct rise over time. 22%

2. I agree with R75 and R76 65%

3. Postoperative imaging is indicated in every case post surgery to serve as baseline

examinations even in patients with undetectable Ct 6%

4. I have no opinion on this matter 7%

Q 10 (n=114)

R78 states that in the absence of residual anatomically identifiable disease (neck US

and CT) in a thyroidectomized patient with a measurable Ct level without previous level VI

LND, an empiric central LND dissection may be considered, but may not be successful.

Do you agree?

1. I agree with the ATA guideline 54%

2. Central LND should be performed, due to a high probability of lymph node metastases

in this compartment even without visible lymph nodes on US. 38%

3. I have no opinion on this matter 8%

Q 11 (n=116)

Postoperative adjuvant EBRT to the neck and mediastinum may be considered in patients

who are found to have microscopic positive margin(s) (R1 resection) following surgery for moderate to high volume disease involving the central compartment (level VI) and one or

both lateral neck compartments (levels 2A-V).

1. I agree with the ATA guideline 53%

2. This recommendation may be accepted only in patients with evidence of incomplete

resection (R2 resection) 22%

3. I do not agree, as EBRT will lead to considerable toxicity without any evidence for

improved overall survival 16%

4. I do not have an opinion on this matter 9%

Q 12 (n=116)

The routine use of cytotoxic chemotherapy should be discouraged in patients with MTC.

It may be considered for selected patients with rapidly progressive disease not amenable

to clinical trials. Do you agree?

1. Yes. 84%

2. No. Cytotoxic chemotherapy is standard of care in patients with metastatic MTC 7%

3. I do not have an opinion on this matter 9%

Q 13 (n=114)

R66 states that in patients with extensive distant metastases a palliative neck operation

may still be needed when there is pain, or evidence of tracheal compromise and the need to maintain a safe airway. Otherwise, in the setting of moderate to high volume extra-cervical disease, neck disease may be observed and surgery deferred (Task Force opinion was not unanimous). Do you agree?

1. Yes 72%

2. No 24%

3. I have no opinion on this matter 4%

Q 14 (n=113)

ATA guidelines do not recommend FDG PET imaging in primary preoperative evaluation.

Do you agree?

1. Yes. 68%

2. No. FDG PET helps in preoperative staging 4%

3. No. Receptor PET imaging is useful in primary MCT staging and should be recommended 4%

4. F dopa seems the best one 18%

5. I have no opinion on this matter 6%

Q 15 (n=120)

Do you agree that FDG PET should be performed in cases of asymptomatic

hypercalcitoninaemia to localize foci of MTC?

1. Yes 31%

2. No. FDG PET is not sufficiently sensitive for detection of small metastatic MTC foci 20%

3. No. FDG PET should be applied to detect metastatic foci only if Ct>400 ng/L 7%

4. No. Due to both 2 + 3 34%

5. I do not have an opinion on this matter 6%

Q 16 (n=117)

Do you agree that MIBG therapy and peptide receptor radiotherapy may be useful in

palliative therapy of advanced MCT

1. Yes. 59%

2. No. 32%

3. I have no opinion on this matter 9%

Q 17 (n=111)

Do you agree with the ATA guideline statement that somatostatin analogues are not

Recommended as antitumor agents in MTC?

1. Yes 38%

2. MCT associated symptomatic diarrhea or Cushing syndrome may be treated with

somatostatin analogues 13%

3. Both 1 and 2 apply 44%

4. I have no own opinion in this matter 3%

Q 18 (n=108)

Do you agree that residual disease as documented by any increase of Ct level without

Localization of the disease should not constitute an absolute contraindication to pregnancy?

1. Yes. 66%

2. No. Pregnancy is contraindicated in any case of persistent MTC, with or without positive

imaging 15%

3. No. Pregnancy may be considered only if doubling time of Ct is less than 2 years 17%

Q 19 (n=102)

ATA guideline R1 recommends RET testing in patients with personal medical history of

primary C hyperplasia in whom no diagnosis of MTC has been made because new carriers

of RET germline mutation can be detected in this way. Do you agree on this procedure?

1. Yes 62%

2. No, because the risk of finding a germline RET mutation carrier is too low 21%

3. No, because this will not be covered by the insurance system 6%

4. I have no opinion on this matter 8%

Q 20 (n=102)

Guideline R10 recommends to consider (Grade A) RET testing in all patients with

Hirschprung disease (HD). Do you agree to test all HD patients in view of HD as a common disease with few RET positive cases?

1. Yes 60%

2. No 6%

3. No, Further research is necessary to disclose the significance of testing for activating

mutations in HD 18%

4. I have no opinion on this matter 13%

Q 21 (n=102)

ATA guideline R11 recommends to perform MEN 2-specific exons of RET (10, 11, 13, 14,

15, 16) as either single or multi-tiered approach. Do you agree?

1. Yes 35%

2. No, RET mutation screening should be completed by exon 8 analysis in all regions

where it was described to be present 23%

3. No. Systematic screening for RET mutations in exon 8, 10, 11, 13, 14, 15 and 16 should

be performed in all patients diagnosed with MTC 26%

4. I have no opinion on this matter 16%

Q 22 (n=95)

If the routine analysis is negative in the clinical setting of MEN 2 or when there is a discre-

pancy between the genotype and phenotype, do you agree with recommendation R12 to

perform sequencing of the entire coding region of RET to identify MTC causative mutations.

1. Yes 46%

2. No. It has a poor cost effectiveness since there are 14 “remaining” exons (1-7, 9, 12,

17, 18, 19, 20, 21) where activating mutations have never been reported 8%

3. No. There is no need to sequence the remaining exons with the exception to look for rare mutations in exon 5 (R321G) 5%

4. 2 + 3 23%

5. I have no opinion on this matter 17%

Q 23 (n=92)

Conflicting results have been published in recent years about the possible role of RET polymorphisms as genetic modifiers, either in sporadic or hereditary MTC. Do you agree,

that ETA comments should include that no definite clinical significance can be given to the presence/absence of RET polymorphic variants and no further research seems necessary

1. Yes 43%

2. No. The data are still insufficient to add this statement 47%

3. I have no opinion on this matter 10%

Q 24 (n=78)

ATA gives in Table 6 a division of RET germline carriers into 4 risk categories (A – lowest, D – highest) which differs from the previous division into 3 risk categories by separation of the

high risk RET 634 and (ATA B class) and moderately high risk of other exon 10 mutations

(ATA A class). Do you agree on this ATA modification?

1. Yes, it rationalizes the differences in phenotype and is clinically relevant 82%

2. No, I prefer the previous division of risk into three categories 10%

3. Yes, but I would prefer a 5 categories division, including the lowest risk RET mutations,

which have not been proven fully (example: RET 649) 8%

Q 25 (n=77)

ATA guideline R2 recommends RET testing of MEN 2B only in cases of intestinal ganglioneuromatosis, because this is often diagnosed before the diagnosis of MEN2B.

Do you agree on this?

1. Yes 32%

2. No, bumby lips with mucosal neuromas should also be considered as indication 13%

3. No, both bumby lips with mucosal neuromas and corneal fibres should also be considered. 55%

Q 26 (n=76)

Ad R2 Do you accept inclusion of the Tearless crying sign as indication for RET testing in small children without any other MEN2 symptoms and with a negative family history?

1. Yes, because it is necessary to diagnose de novo MEN2 cases as soon as possible 34%

2. No, the prevalence of tearless crying is not well defined in normal children and there is a

high risk of false results 25%

3. No, more data are necessary. However, I find this sign worth mentioning in the comments. 41%

Q 27 (n=78)

The ATA R4 recommends RET testing in cases of lichen planus (Grade B). The proposal is

to strengthen this recommendation: Lichen planus amyloidosis or pruritus in the central upper

back may indicate the presence of a RET mutation and should prompt Ct measurement in

adults and genetic testing in children. Do you accept this proposal?

1. Yes. 62%

2. No, I prefer the more cautious standpoint of the ATA Guidelines 16%

3. I propose to mention it in the comments but not change the ATA R4 recommendation. 20%

Q 28 (n=80)

ATA guidelines (R6-8) recommend to consider ATA risk class and patient’s age and allow to

“delay prophylactic thyroidectomy beyond age 5 years in patients with ATA Level A and B

RET mutations in the setting of a normal annual basal +/- stimulated serum Ct, normal

annual neck US, less aggressive MTC family history and family preference”. “For higher risk mutations consider treatment before age 5 in an experienced tertiary care setting”.

1. I accept this statement fully 61%

2. Prophylactic thyroidectomy should be done when stimulated Ct starts to rise 26%

3. Prophylactic thyroidectomy should be done before Ct starts to rise at the age of 3-5 years 8%

4. I have no opinion on this matter 5%

Q 29 (n=84)

The ATA guidelines state that there is rarely a need for stimulated Ct testing for deciding on prophylactic Tx. Do you agree?

1. Yes. It is a burden for a young patient, with a risk of not being cured after surgery. 31%

2. Yes, in addition to 1, sensitive Ct assays have to prove their value in these circumstances 25%

3. No. Stimulated Ct helps to define the optimal time point for prophylactic Tx. 37%

4. I have no opinion on this matter 7%

Q 30 (n=90)

When prophylactic thyroidectomy is delayed beyond fifth year of life, Ct should be measured:

1. every 6 months 36%

2. every year 52%

3. every year until the age of 20, then every 2-3 years 12%

Q 31 (n=84)

Ad R6-8. Please select the level of Ct allowed to delay the prophylactic/preemptive

thyroidectomy in ATA class A and ATA class B RET mutation carriers, who have less

aggressive family history

1. At normal basal Ct (≤10 ng/L) and normal stimulated Ct (≤ 30 ng/L) 45%

2. At normal basal Ct (≤10 ng/L) and only slightly elevated stimulated Ct (≤50 ng/L) 20%

3. At normal basal Ct (≤10 ng/L) and only moderately elevated stimulated Ct (≤ 100 ng/L) 12%

4. I do not see the role for stimulated calcitonin estimation in this setting, evaluation of

basal Ct is sufficient 17%

5. I do not have an opinion on this matter 6%

Q 32 (n=82)

Do you agree to complete ETA comments with the statement: It is also of high importance,

that high volume surgeons perform pre-emptive thyroidectomies with or without additional

lymph node dissection

1. Yes 39%

2. No, children should be operated only by paediatric surgeons 0%

3. It depends on the experience of the given centre, but prophylactic/preemptive Tx should

be performed only in experienced tertiary reference centers 57%

4. I do not have an opinion on this matter 4%

Q 33 (n=77)

The ATA R20 recommends preoperative Ct testing in children-RET carriers with the

exception of the MEN2B carriers younger than 6 months old. Do you agree?

1. Yes 59%

2. No. It is unclear why children younger than age 6 months should not have Ct assessment. 15%

3. Reference ranges for small children are not well set, thus prophylacticTx should be as

early as possible and independent of Ct level 24%

Q 34 (n=65)

R38 and R42 state that in asymptomatic MEN 2A and FMTC patients who present at age

>5 years and asymptomatic MEN 2B patients who present at age >1 year, further evaluation

prior to surgery, and more extensive surgery is needed if basal serum Ct is >40 ng/L, if thyroid nodules are > 5 mm, or if suspicious lymph nodes are identified on neck US. Do you agree?

1. Yes 57%

2. Preoperative basal and stimulated Ct and thyroid sonography are always necessary to decide whether resignation of central LND is possible 25%

3. Cut-offs of basal Ct>40 pg/ml and thyroid nodules of >5mm may be regarded as cut-off

between prophylactic and therapeutic Tx but not define extent of surgery 18%

Q 35 (n=80)

The ATA guidelines recommend screening for hyperparathyroidism (PHPT) in asymptomatic

RET mutation carriers by 8th year of age in ATA C (634 and 630) RET mutation carriers and

by 20th year of age in other RET mutations associated with MEN2A. Do you agree?

1. Yes 67%

2. No, 8 years is too early as there are few cases of PHPT before the 3rd decade, age 20

years is more appropriate if no specific family data 28%

3. I have no opinion on this matter 3%

Q 36 (n=80)

ATA R49-50 guidelines recommend surgical treatment for PHPT in MEN2A, preferred to

medical therapy, in the absence of contraindications such as excessive surgical risk or

limited life expectancy. Do you agree?

1. Yes 52%

2. The optimal surgical management of HPT in MEN2A is not yet defined. Decision for

surgery for mild HPT should be assessed per individual patient 42%

3. I have no opinion on this matter 5%

Q 37 (n=73)

The ATA guidelines recommend that screening for PHEO is necessary by 8th year of age in

ATA D class (MEN2B ) and ATA C (634 and 630) RET mutation carriers. Do you agree?

1. Yes 32%

2. Age of 8 years seems very early, only few cases of PHEO before 3rd decade, age18

years is more appropriate unless specific family data on earlier appearance 11%

3. Always if an operation or pregnancy is planned 11%

4. 2+3 42%

5. I have no opinion on this matter 4%

Q 38 (n=73)

In MTC diagnosed/suspected preoperatively in which the familial anamnesis is negative

if no RET data are available prior to surgery, ATA R53 guideline recommends at least one

test – biochemical or CT/MRI. What is your opinion?

1. PHEO biochemical screening is mandatory in any case of suspected MTC. 33%

2. CT/MRI imaging is an alternative to exclude an adrenal tumour 16%

3. 1+2 are necessary 47%

4. No additional investigations are necessary in the absence of hypertension 4%

Q 39 (n=74)

ATA guidelines recommend that PHEO should be surgically resected after appropriate preoperative preparation and prior to surgery for MTC or PHPT. ATA R54-55 and R60

do not require scintigraphy to exclude extraadrenal pheo/malignancy. Do you agree?

1. Yes I agree with ATA guideline because the risk for extraadrenal pheo is extremely

small in MEN2A. 58%

2. No, scintigraphy should be included in the preop assessment of PHEO in MEN2A to

exclude rare cases of malignancy/multiple pheos. 35%

3. I have no opinion on this matter 7%
